# Where do they come from and where do they go: Understanding the relationship between deprivation and the geographical journeys of trainee doctors in England

**DOI:** 10.1371/journal.pone.0345301

**Published:** 2026-03-30

**Authors:** Barry Rowlingson, Peter Diggle, Liz Brewster, Michael Lambert

**Affiliations:** 1 Centre for Health Informatics, Computing, and Statistics (CHICAS), Lancaster Medical School, Lancaster University, Lancaster, United Kingdom; 2 Lancaster Medical School, Lancaster University, Lancaster, United Kingdom; University College London, UNITED KINGDOM OF GREAT BRITAIN AND NORTHERN IRELAND

## Abstract

Having enough doctors to provide healthcare services is a concern internationally. In the UK, significant resources for education and training have been devoted to medical workforce management. Nevertheless, some areas of the country still struggle to recruit and retain staff compared with others. Solutions to this problem have focused on attracting students from backgrounds not traditionally represented in medicine to choose it as a career, and opening new medical schools in different areas of the country. The main objective of this paper is to examine medical student and doctor distribution in order to contribute to understanding the distribution of health and health service inequalities. We used a modelling approach to understand characteristics of medical students, medical schools and foundation schools to interpret and identify the relationship between geographic distribution and socio-economic deprivation. This geographical and statistical analysis aims to identify patterns in workforce distribution, layering these with data on deprivation and inequality. Analysis shows that there are fewer students who come from from more deprived areas, and that different patterns can be observed in geographic locations of training when considering gender and ethnicity. While there is greater diversity of the future workforce in terms of gender and ethnicity, there is evidence that fewer students from more deprived backgrounds are attending medical schools. This has implications for the future workforce, and medical schools may need to play a greater role in increasing access to medical education to overcome observed inequalities.

## 1 Introduction

In the United Kingdom (UK), evidence suggests that access to care is not equitable, and this contributes to wider inequalities in health outcomes [1]. Some of these inequalities in access are caused by workforce shortages. Intervention is focused on the recruitment and retention of medical staff vital to maintaining services [2]. Often this intervention concentrates on the start of the training pathway, aiming to diversify and expand the number of medical students who will subsequently join the workforce. This paper aims to consider workforce distribution, in relation to medical training pathways, with attention to health and healthcare inequalities. We need to understand the origins of medical students, their progression through the training pathway and how demographic differences may shape choices made by medical students and doctors, in order to consider how to redress issues identified in unequal access to care.

Medical workforce distribution and access to healthcare are also topics of international relevance and concern [3]. Despite widespread attempts to ensure a more equitable distribution of doctors, it remains a concern both between countries (the ‘brain drain’ of medical professionals from the Global South to the Global North [4,5]) and within countries, with remote/rural areas as presenting particular staffing challenges [6,7]. This distributive issue is an ethical one in Global North countries like the UK, with NHS Employers highlighting a list of ‘red countries’ from which medical staff should not be recruited, based on the World Health Organisation’s Workforce Support and Safeguard List [3,8].

Recent UK policy planning states that ‘self-sufficiency’ of supply of doctors is an ambition for medical workforce management. The goal is to reduce reliance on international recruitment of doctors, and with this comes a commitment to rapid expansion of medical school training places [2]. While the proposed plan is currently under review [9] the stated ambition to double the number of medical students from 7,500 in 2022–15,000 by 2031/32 shows its scale. This approach of increasing numbers of medical students to solving the workforce crisis is not unique to the UK. Policy initiatives often focus on new medical graduates, who have the potential to contribute to the workforce for longer, and are also more amenable to intervention (e.g., by increasing the number of funded or subsidised medical school places as above).

Overall, the Organisation for Economic Co-operation and Development (OECD) estimates that the number of medical graduates across OECD countries has increased to 160,000 in 2021, up from 93,000 in 2000 [10]. While this is a particularly positive rise if increasing the number of medical graduates is positioned as a solution to the issues faced in access to healthcare, there remain inequalities between countries. The UK has a lower number of doctors than comparable OECD countries, with 3.18 per 1000 population compared with the OECD EU average of 3.94 and fewer medical graduates per 100,000 population estimated at 13.1 per 100,000 population compared with the OECD 36 average of 14.2 per 100,000 [10,11]. There are also inequalities within countries as some areas (e.g., remote and rural locations) struggle with recruitment of workforce more than others.

Increasing the number of medical students overall goes only part way to solving the problem of access to healthcare. There is also a need for any increase in student numbers to be sustained with an increase in post-graduation training places. Caps on the number and distribution of medical school places are managed separately from postgraduate training places. However, there is recognition that the distribution of postgraduate training places is also an issue. Distribution patterns do not match population need, and are an artefact of previously-established systems, which leads to issues in access to healthcare in particular parts of the UK [12]. Recent plans to revise the organisation of medical training acknowledges the impact of geography, stating that ‘medical training posts have been distributed across England based on historical arrangements and this has not fully aligned with the current or future health needs of local populations’ [13]. Although efforts are being made to overcome some of these challenges of workforce distribution, others are critical of current strategies, which do not provide a robust package of measures that focus on improving workforce development via broader incentives (e.g., initiatives that would reduce inequalities within communities such as greater access to high-quality education) [14,15].

This recognition of geographic inequality in postgraduate training has also driven initiatives in undergraduate education. Over the past 20–25 years, new medical schools have been founded on the justification that future doctors will stay where they are originally educated and trained. This justification acknowledges a specific goal for newer schools to work locally in areas that are currently underserved. The admissions and selection process at medical schools is independent from this longer term goal, though some medical schools explicitly state their “specific aim of improving the recruitment and retention of doctors” in their location [16].

Some medical schools throughout the UK commit to improving access to medical careers for applicants whose backgrounds have been under-represented in the profession, often under the banner of ‘widening participation.’ The aim within these schemes is to increase the number of medical students overall, but also to diversify professional demographics. In medical education, two trends have shaped subsequent widening participation policies. First, recognition that doctors should better reflect patient communities served, requiring changes in medical school admissions policies [17]. Second, recognition that efforts to widen participation in medicine lagged behind national trends in other areas [18]. This prompted the Medical Schools Council [19], the representative body for medical schools in the UK, to issue guidance on best practice for enacting and monitoring widening participation initiatives in medical school admissions processes. Universities retained extensive discretion to define and measure both widening participation, and the outcomes of changed policies and practice.

Priority groups for widening participation are independently set by each medical school. Several new medical schools (founded in the post-2017 expansion of student places), including Lincoln, and Kent and Medway, concentrate upon recruitment and selection from, and service for, their local underserved populations [16,20]. Elsewhere, other universities prioritize students who are the first in the family to go to university and those from a community where low numbers attend university [21], or look to eliminate gaps identified by the medical school in rates of offering interviews to particular applicant groups [22]. This ‘pick and mix’ approach reflects the ‘best practice’ framework [19] and allows universities latitude within broad parameters. However, there is still a persistent under-representation of students from deprived backgrounds in medicine [23].

Medical workforce planning requires a detailed understanding of population healthcare need. We view the geography of healthcare provision as interlinked with medical schools, despite their separate drivers in policy. For example, newer medical schools are not co-located with hospitals, which has implications for recruitment of clinical academic staff and clinical placement provision. This then leads to differences between medical schools, which we expand on below.

Medical workforce distribution and health inequalities have a bidirectional relationship. A lack of medical workforce causes a lack of access to healthcare for the population, which has a negative impact on patient health outcomes. These healthcare inequalities lead to increased workload associated with managing patients with poorer health outcomes [24,25]. Inequalities therefore also have an impact on distribution of medical workforce, as some areas of the country are less desirable to live and work in. These factors make it harder to recruit and retain medical staff in some areas. Health inequalities are known to be distinctly regional, with healthy life expectancy and quality of life declining in the most deprived areas [26]. Unequal distribution of resources leads to inequalities of health outcomes including morbidity and mortality. Access to affordable, quality healthcare is also one of the social determinants of health [27].

Evidence shows that health issues are more prevalent in areas of socio-economic deprivation and that structural inequalities increase the risk of developing health problems [26,28]. People living in the most deprived areas of the UK are more likely to die from avoidable causes, have long-term health conditions and a higher disease burden than those in less deprived areas [1,29]. The social determinants of health, including income, employment, housing, and environment are the main causes of these poorer outcomes. Alongside these multiple factors there is a need for a robust, cohesive healthcare system to support patients. If appropriate and timely care is difficult to access, then patients do not receive preventative care or treatment meaning that the disease burden and associated avoidable mortality are higher. Health inequalities have increased during the recent pandemic, further perpetuating the issue [30].

This paper contributes to our understanding of the distribution of health inequalities by examining medical workforce distribution through the first two transitions along doctors’ training pathway, from home to medical school and from medical school to foundation school. We provide a geographical and statistical analysis to identify recent patterns in the workforce distribution, layering these with data on socio-economic inequalities.

## 2 Materials and methods

### 2.1 Data sources and dataset construction

#### 2.1.1 The UK medical education database.

The UK Medical Education Database (UKMED) is a collation of datasets that provides a unique overview into recent patterns in medical education and training. Combining data from higher education, postgraduate training bodies and the General Medical Council as medical regulator, it provides detailed anonymised data on all those who have started a medical degree since 2002. This makes it a valuable resource to interrogate to understand patterns of workforce distribution. Our extract was an update of UKMEDP044, previously generated in 2018 to cover 2002−2015, updated to include 2016−2022. Data were accessed for research purposes (14/12/2023). Ethical approval was granted by Faculty of Health and Medicine Research Ethics Committee Lancaster University on 22 August 2022 (ref: FHM-2022–0970-IRAS-1). As this was a retrospective study of an existing dataset, participants did not provide informed consent to the research team, and the authors did not have access to information that could identify individual participants. The use of personal data in UKMED is not reliant on individual consent from data subjects and UKMED data are only disclosed to researchers in a pseudonymised form through a Safe Haven which prevents identification (see ‘data source’ for full justification of consent waiver).

Our analysis of the UKMED data aims to understand the potential impact of deprivation on workforce distribution, and uses data from students awarded their medical qualification between 2004 and 2022, allowing us to account for some of the more recently established medical schools (post-2017). The dataset contains anonymised individual student information on their temporal, geographical, academic and professional journey from family home to medical school and from medical school to postgraduate places of work as a resident doctor. While UKMED data allows for individual level analysis, we wanted to consider where medical students did not come from (not in UKMED data), as well as where they did come from. Our interest was in understanding whether the deprivation of the LAD affected the potential number of medical students.

The required information for our analysis included the distances of the various career steps (e.g., from home to medical school; medical school to foundation programme training; foundation programme to specialty training) as well as demographic and socio-economic data [31]. From these records we constructed two sets of counts of students for modelling two transition stages in medical education: from home to medical school, and from medical school onwards to foundation school. As we are interested in understanding the impact of more recent changes in medical education, we have not modelled foundation to specialty training transitions, as there are fewer students in these data, and none from the newer medical schools.

The UKMED data extract supplied contains approximately 157,000 individual records. For our modelling purposes we formed two subsets, one for home local authority to medical school, and one from medical school to foundation training.

#### 2.1.2 Home (local authority) to medical school dataset.

We modelled three measures of the number of students from home to medical school: the total number of students, the number of female students, and the number of non-white ethnicity students. Home was defined as the local authority district (LAD) recorded. UKMED provides both gender and ethnicity to enable classification and counting the number of students in these categories moving from any LAD to any medical school.

Because of statistical differences in census and deprivation measures across the UK, we restricted our analyses to students with residence in English local authorities going to English medical schools only. We used LAD geographies for 2019, resulting in 317 spatial units. The parental home postcode field from UKMED was used to assign a LAD to a student’s initial location. This was also used for graduate entry students (around 15% of the total) who may not have lived at their parental home for some time, but we considered that it best represented their home/ origin location.

Only medical schools offering a full medical degree-level qualification were included in analyses, meaning universities only offering a one-year gateway course (e.g., the University of Bradford) were excluded. These medical schools represent accepted applications as collected in Higher Education Statistics Agency (HESA) data, (it is possible, but less common, to change medical school following initial enrolment). This selection results in 35 medical schools, accounting for mergers and splits over time (e.g., Peninsula becoming separate schools at Exeter and Plymouth, mergers in London schools over time). Our dataset was then a table of 11,095 rows giving the count of medical students from each of the 317 LADs going to each of the 35 medical schools. We augmented this table with local authority data, medical school data, and data relating to individual LAD to medical school pairs.

Data from the UK government Office for National Statistics (ONS) was used to obtain population totals at the LAD level from the 2021 census data. Additionally we used the single year-of-age population count data to get the fraction of the population aged between 13 and 17 to model the typical age demographic of those considering applying to medical school. We also used a coarse ethnicity measure to get the fraction of population that identify as non-white in each LAD. This is a highly simplistic approach to ethnicity, so our conclusions will be similarly simplistic. However, a white/non-white dichotomy represents the broadest division in UK society in terms of the medical profession and widening participation. We recognise and acknowledge the wider issues with race and inequalities that may be obscured by this approach [32].

As a simple exploratory step we computed the number of medical students in the dataset coming from each LAD relative to the 13–17 year-old population size. This was then tabulated and mapped over the country to get a sense of any broad spatial trends in the origin of medical students.

We used the 2019 English Indices of Deprivation data which form the Index of Multiple Deprivation (IMD) for each LAD. As part of the analytical process, we reviewed the seven components of this index on an individual basis, to consider inclusion in modelling as individual elements in the ensuing models. Having established that individual IMD element modelling did not contribute more than overall IMD, we only use and report IMD here.

We categorised medical schools by the era in which they were founded, accounting for the impact of regulatory, health services and higher educational policy relations on medical training. Five categories were identified across the UK (English and Scottish Historic, British Civic, British Young Civic, Plateglass and Marketised). This novel classification, designed by an NHS and social policy historian (ML), represents an attempt to look beyond individual medical schools to consider a clustering of medical schools by wider characteristics. These characteristics include their foundation, size, location, relationship to hospital construction and rationalisation in the NHS, and the university with which they are associated. Each of these has developed over time and influence on the culture and character of the school and the type of doctors they produce. Not all medical schools in this table are represented in the dataset as we focus only on England in this paper, not the whole of the UK, but are listed for completeness with further details in supplementary material S1 File Information).

The geographic data for LAD boundaries included a population-weighted centroid, and we used the straight-line distance from this centroid to the principal site of the medical school (measured using Ordnance Survey Grid Reference coordinates) as the distance between LAD and medical school. Our resulting data set for modelling initial medical school choice with 11,095 rows had columns as summarised in Table 1.

**Table 1:** Data used to model home local authority district to medical school.

*i*	Local Authority District (LAD) identifier
*j*	Medical school identifier
*Y*_*ij*_	Number of students from LAD *i* to medical school *j*
$Y_{ij}^{\text{F}}$	Number of female students from LAD *i* to medical school *j*
$Y_{ij}^{\text{NW}}$	Number of non-white students from LAD *i* to medical school *j*
POP_*i*_	LAD population from the 2021 census
$f_{i}^{\text{age}}$	Proportion of the LAD population in the age 13–17
ETH_*i*_	non-white LAD population proportion from the 2021 census
IMD_*i*_	Index of Multiple Deprivation score for the LAD
ERA_*j*_	Medical school era classification (see supplementary material S1 File Information)
*d*_*ij*_	Distance from population-weighted centroid of LAD *i* to medical school *j*

Continuous explanatory variables ETH_*i*_, IMD_*i*_ and *d*_*ij*_ were scaled to be zero-mean and unit variance to compare relative effect strengths across variables.

This dataset represented around 101,000 students. The total number of students from a LAD ranged from under 5 for the Isles of Scilly to over 2,500 for Birmingham, with a mean of around 320 and a median of 230. This skewness is primarily a feature of the skewness in the populations of LADs, which range from around 10,000 to over 1,000,000. The total number of students going to each medical school varied with the size and age of the medical school, with Edge Hill having only 50 students in the data, and Birmingham having over 6,500. The number of students going from a LAD to a medical school ranged from 0 to 736, with a mean of around 9, a median of 4, and a modal value of zero. The gender proportions were 42% male, 58% female, and the ethnicity proportions were 58% white, 42% non-white.

#### 2.1.3 Medical school to foundation school dataset.

Foundation training represents the first two years of employment in the NHS after graduation from medical school. Resident doctors (formerly “junior doctors”) achieve full registration as doctors at the end of the first year of foundation training. It is organised geographically, in nominal “Foundation Schools” (training areas). The boundaries of these training areas in England have been subject to many organisational changes during the period of our data extract. The UKMED dataset contains 81 unique organisation names identifying foundation school iterations.

To overcome this, as we were interested in locational aspects of training choices, we classified medical students’ foundation training into one of 12 geographical areas across England. We constructed a lookup table of these 81 names to 12 areas to map any individual movement from medical school to foundation school training to one of these 12 areas. These were named after the Foundation Schools and/or Postgraduate Deaneries that best represent these areas, although in some cases, these areas are amalgamations of previous and extant organisational units (Table 2).

**Table 2:** Foundation school areas, defined to take into account change over time.

• London	• Kent, Surrey and Sussex	• West Midlands	• Yorkshire and Humber
• North West	• Severn	• East Midlands	• Wessex
• Peninsula	• Northern	• Oxford	• East of England

Nine medical schools had fewer than ten medical students progressing to foundation training in the dataset, mainly as students had not yet graduated from the five-year course, and so were removed from this analysis. 27 medical schools were therefore included in analysis; when combined with the 12 foundation school areas this provided 324 counts from medical school to foundation school for modelling. As with the LAD to medical school step, we used the total number of students going from each medical school to a foundation school, the total number of female students, and the total number of non-white students to look at aspects of gender and ethnicity in foundation training. The total number of students represented in this stage of the analysis was 61,575. The gender proportions were 44% male and 56% female, and the ethnicity proportions were 63% white, 37% non-white.

Foundation schools, as defined, are large and heterogeneous areas, and so it was not possible to construct useful measures of deprivation or population demographics as we did for LADs. Medical schools, based in universities, are located within these broad foundation schools, but are not connected to them organisationally.

Given the difficulties in differentiating between foundation schools in terms of deprivation or demographics, we sought additional measures to model. The UK Foundation Programme website publishes “competition ratios” (CRs) for foundation schools. These CRs show the most and least subscribed areas, and essentially provide data on the popularity of an area compared to the number of available places. They are constructed from the number of first choice applications to the foundation school programmes divided by the total number of available programmes in the foundation school area. Hence, a CR above one means there were more people choosing the location as their first choice than available places, and a number below one means that area had fewer first choices than places. We used the CR data for 2021–2024, merged to map to our 12 areas, to give us a numeric competition ratio, *c*_*j*_, for use in our model. Although we used UKMED data covering a broader period (2002–2022) we expect that areas have remained roughly similar in popularity.

The UKMED data included the postcode of individual foundation school placement locations. To compute a representative point location for each of our 12 areas, we took an average of all the postcode locations of placements within each area. These locations were then used to construct two distance measures for each medical school to each foundation school:

- *d*_*ij*_, the geographic distance in km measured as a straight line from medical school *i* to foundation area *j* point location.
- *s*_*ij*_, a discrete measure from medical school *i* to foundation school *j* with three values: “Local” if *j* is the coterminous (i.e., the medical school is located within the foundation school to *i*), “Neighbour” if *j* is the next-nearest foundation area, and “Distant” if *j* is any other foundation area. The total number of medical students in each of these pseudo-distance categories is given in Table 3.

**Table 3:** Number of medical students going to foundation schools by distance categories (rounded to nearest 5).

Category	Total
Local	27150
Neighbour	6630
Distant	27795

In addition, given the position of London as the economic centre of the UK and its high number of students within five medical schools we also separately reviewed data from London, in order to investigate any potential effect. London schools, which have changed over time are at time of writing: King’s College London [short name: King’s], University College London [UCL], Barts and The London School of Medicine and Dentistry, [short name: Barts], Imperial College London [short name: Imperial] and City St George’s, University of London [short name: St George’s]). We constructed a four-level categorical factor that classified whether the move from medical school to foundation school is wholly within London, from a London medical school to a non-London foundation school, into a London foundation school from a non-London medical school, or from a non-London medical school to a non-London foundation school. Table 4 shows the total number of students whose medical school to foundation school moves are in each of these categories.

**Table 4:** Number of medical students moving to a foundation school by relationship with London (rounded to nearest 5).

Category	Total
Non-London	39430
Out of London	11460
Into London	4985
Within London	5700

Since the total numbers going from each medical school and the total numbers of students going to each foundation school are only dependent on the capacity of these organisational units, they are not of intrinsic interest in modelling except as scale factors of numbers of students, to appear as an offset term in models. Hence we computed *E*_*ij*_


$$E_{ij}=f_{i}^{M}f_{j}^{F}N$$ (1)

where $f_{i}^{M}$ is the overall fraction of the students from medical school *i*, $f_{j}^{F}$ is the overall fraction of the students going to foundation school *j*, and *N* is the total number of students in the dataset. These *E*_*ij*_ values are then the expected numbers from medical school *i* to foundation school *j* under an assumption of completely proportional movement of the total number of medical school students. Table 5 summarises the variables for modelling the medical school to foundation school analysis.

**Table 5:** Data used to model medical school to foundation school.

*i*	Medical school identifier
*j*	foundation school identifier
*Y*_*ij*_	Number of students from medical school *i* to foundation area *j*
$Y_{ij}^{\text{F}}$	Number of female students from *i* to *j*
$Y_{ij}^{\text{NW}}$	Number of non-white students from *i* to *j*
ERA_*i*_	Medical school era classification
*c*_*j*_	Competition ratio for foundation area *j*
*L*_*ij*_	London-movement factor
*d*_*ij*_	Distance from medical school *i* to foundation area *j*
*s*_*ij*_	Discrete distance measure from *i* to *j*
*E*_*ij*_	“Expected” value of *Y*_*ij*_ under proportional movement

### 2.2 Model formulation and fitting

We used a generalised linear modelling (GLM, [33]) framework to fit three models of student movement numbers over the two educational transitions for a total of six final models. For each transition we fitted the total number of students moving from source unit *i* to destination unit *j*, as well as a measure to see if there were differences in the training patterns between female/male students and non-white/white students. For the total number of students models, we used a negative binomial response regression model, with a log-linear term. Our model for the number of students from source *i* to destination *j* was then:


$$\log(\mu_{ij})=\log(N_{ij})+\beta X$$ (2)

$$Y_{ij}\sim \text{NB}(\mu_{ij},\theta )$$ (3)

where *N*_*ij*_ is an offset used to offset the sizes of sources or destinations, β is a vector of parameters and *X* is a matrix of explanatory variables. NB(μ,θ) is the negative binomial distribution with mean μ and overdispersion θ.

For the female/male and non-white/white comparison models we looked at the difference in the ratios of the number moving from *i* to *j* in each class as a fraction of the source unit population of that class in each source *i* unit. For example, given $n_{ij}^{w}$ women and $n_{ij}^{m}$ men going from *i* to *j*, where the number of women in *i* is $N_{i}^{w}$ and the number of men is $N_{i}^{m}$ we modeled the quantity $R_{ij}^{d}$


$$R_{ij}^{d}=\frac{n_{ij}^{w}}{N_{i}^{w}}-\frac{n_{ij}^{m}}{N_{i}^{m}}$$ (4)

as a Gaussian linear model with the same explanatory variables as in the negative binomial model. Local authorities were approximately gender-balanced within the age group we used, so we simplified the scaling to be the source population size. For ethnicity models we used the census population ethnicity fraction to scale the denominators in Eq 4 appropriately.


$$\eta_{ij}^{s}=\beta X$$ (5)

$$R_{ij}^{d}\sim \text{Norm}(\eta_{ij}^{s},\sigma^{2})$$ (6)

We used a different set of explanatory variables for each of the transitions, since they applied to different sets of administrative units. Models were fitted using the glm function in the R stats package [34] for linear models and the glm.nb function from the MASS [35] package for the negative binomial models.

#### 2.2.1 Home to medical school.

It is reasonable to expect a LAD with a large population to send more students to medical school than a small one. Medical education in the UK typically starts at school-leaver level, usually age 18, though there are some schools which only accept graduate entry students with a first university degree. Given that the majority of students are in the school-leaver category, a better assumption is that LADs with large *young* populations will send more students to medical school than a similar sized LAD with an ageing population. To account for this we used $\log({\text{POP}}_{i}\cdot f_{i}^{\text{age}})$ as an offset term in the model. To account for the size of medical schools, we added a categorical term to the model with a level for each medical school ($\phi_{j}$). The fitted parameter estimates for these levels then accounted for variation due to the total medical school capacity.

Three explanatory variables of interest were identified, as per Table 1:

- *d*_*ij*_, the distance from LAD to medical school;
- ETH_*i*_, the fraction of non-white population in the LAD;
- IMD_*i*_, the index of multiple deprivation for the LAD.

Each of these was modelled as an interaction term with the medical school, which determined if each medical school’s relationship with the explanatory variable differed either from a null relationship or between themselves. We used the medical school era category for visualisation only.

Likelihood-ratio testing of step-wise addition of explanatory variables showed them all to add value when included in the model. Our model for $\mu_{ij}$ in Eq 2 for the number of students moving from home LAD *i* to medical school *j* was:


$$\log(\mu_{ij})=\log({\text{POP}}_{i}\cdot f_{i}^{\text{age}})+\phi_{j}+\alpha_{j}{\text{ETH}}_{i}+\beta_{j}{\text{IMD}}_{i}+\gamma_{j}d_{ij}$$ (7)

For the models for the gender and ethnicity subsets we used the same explanatory variables and the specific formula for Eq 5 was:


$$\eta_{ij}=\alpha_{j}{\text{ETH}}_{i}+\beta_{j}{\text{IMD}}_{i}+\gamma_{j}d_{ij}$$ (8)

since models for proportions do not need to explicitly account for the size of the LAD or school.

#### 2.2.2 Medical school to foundation school.

As the number of students going from a medical school to a foundation school is fixed and the quantities of are not of intrinsic interest, we used the number under a simple proportional model, *E*_*ij*_ in Table 5, as an offset term.

For explanatory variables, we used the foundation school competition ratio to investigate if proportionally more or fewer students from different medical schools were allocated to more competitive foundation schools by including this as an interaction term between medical school and competition ratio.

A log-likelihood ratio test showed the discrete distance measure *s*_*ij*_ to be better than the linear distance term *d*_*ij*_ in the model so we used this term for modelling. We included the intra/extra-London factor *L*_*ij*_ to investigate whether London had a strong influence on medical school choices that could be observed in foundation school choices/allocations. Our model for the number of students going from medical school to foundation school was:


$$\log(\mu_{ij})=\log(E_{ij})+\alpha_{i}c_{j}+\beta s_{ij}+\gamma L_{ij}$$ (9)

and for the gender and ethnicity subsets was:


$$\eta_{ij}^{s}=\alpha_{i}c_{j}+\beta s_{ij}+\gamma L_{ij}$$ (10)

Denominators for computing $R_{ij}^{d}$ in Eq 4 were taken as the total number of students in the medical schools in the relevant gender and ethnicity classes. Since the difference in the gender and ethnicity ratios have no *a priori* scaling with the size of organisational units, unlike total counts in the model of Eq 9 there is no need to include a term like *E*_*ij*_ in the model. Also, since the competition ratio is the only continuous explanatory variable in this model, there is no need for scaling before fitting as we did for continuous explanatory variables in the models for home to medical school.

## 3 Results

### 3.1 Transition from home to medical school

**Fig 1:**
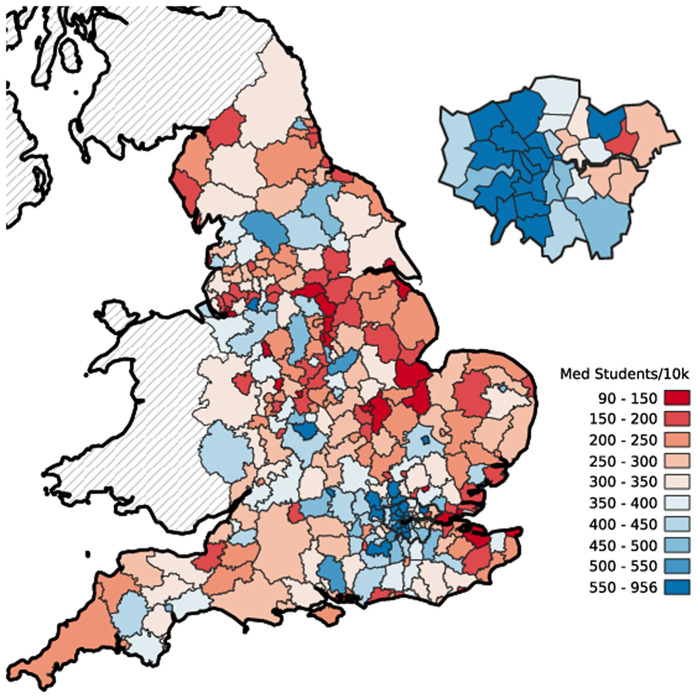
Medical school students per 10k population from LADs with London expanded for clarity. Boundary data: Source: Office for National Statistics licensed under the Open Government Licence v.3.0; Contains OS data © Crown copyright and database right 2019.

Fig 1 shows the number of medical students in the dataset from each LAD per 10,000 people in the 13-to-17 age group, with London expanded for clarity. The total number of students in the dataset per 10,000 total population is 316, so the map is shaded with this as a central neutral colour. Red shows areas with fewer than this many students, blue shows those with more. The map shows spatial structuring that does not appear to be random. For example, comparing West and East London shows that more students are coming from West London than East.

Table 6 shows the top and bottom five LADs in this map. At the top, Harrow has nearly three times the number of students expected in the dataset given its population size, and at the bottom Corby has nearly one-quarter of the number of students. This table and map, coupled with knowledge of the demographics of the local authorities, provide a *prima facie* case for formal statistical modelling to incorporate demographic information such as the deprivation index data.

**Table 6:** The five local authority districts with highest and lowest medical school student/population ratios.

Rank	LAD name	Medical students/10k population
1	Harrow	956
2	Cambridge	941
3	Kingston upon Thames	810
4	Barnet	792
5	Oxford	784
(307 rows omitted)		
313	Kingston upon Hull	109
314	Thurrock	108
315	Swale	106
316	Fenland	103
317	Corby	90

**Fig 2:**
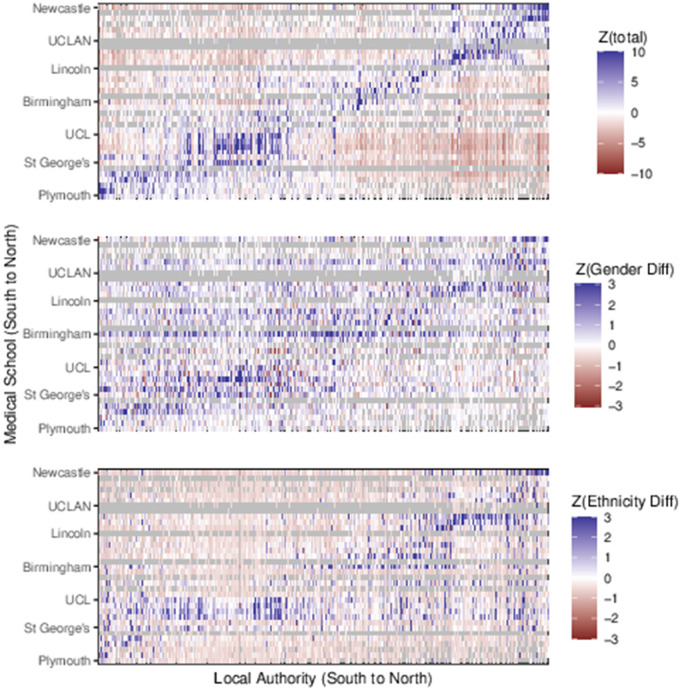
Home to medical school response data ordered by south-north location; Top: Total Number of students; Middle: Gender difference (F-M); Bottom: Ethnicity difference (White-NonWhite). Values Scaled and Centred.

Fig 2 shows approximate geographic structure in the response data for each of the fitted models. Each panel is a grid of LADs (unlabelled) on the X-axis, and medical schools on the Y-axis. LADs and medical schools are ordered south-to-north on their axis. A subset of medical schools are labelled to provide an idea of the south-north trend.

The top panel shows, for LAD *i* and medical school *j*, the value $Z(i,j)=(Y_{ij}-E_{ij})/{\sqrt{E}}_{ij}$ as defined in Table 1. Values are capped at ±10 to avoid outliers stretching the colour scale. When zero students have gone from a LAD to a medical school, the grid is coloured grey. If there was no location preference in medical school attendance we would expect this grid to be unstructured noise with a unit standard deviation. The strong blue diagonal shows an excess of students attending nearer medical schools, although this is confused slightly by the east-west spread of the country and is illustrative only. Our analysis uses a true distance measure from LAD to medical school.

The middle panel is similar and shows the scaled difference in gender ratio (female minus male) for movements from LAD to medical school as described in Eq 4. The diagonal effect here indicates that perhaps female students do not travel as far as their male counterparts, although the effect is not as strong as in the first plot and the overall effect is more of noise. The bottom panel shows the scaled difference in ethnicity ratio as described in Eq 4. The diagonal effect is even less clear here, especially in the London and northern (top right) sector.

**Fig 3:**
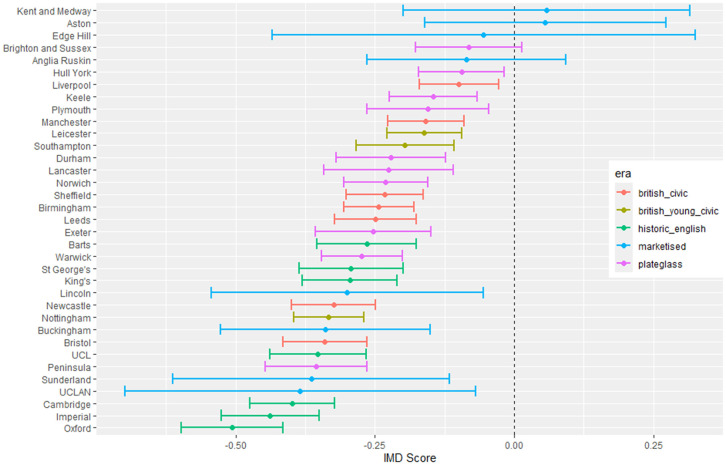
Medical school interaction effects for number of students with increasing local authority deprivation index (IMD score).

The outputs for interpretation from the fitted models are shown from Fig 3 to Fig 13, and consist of maximum-likelihood estimates and standard errors of the regression parameters for each medical school. We present these as charts with parameter value on the X axis, and medical school on the Y axis. A 95% confidence interval (from ±2 standard errors) is displayed as a horizontal bar.

**Fig 4:**
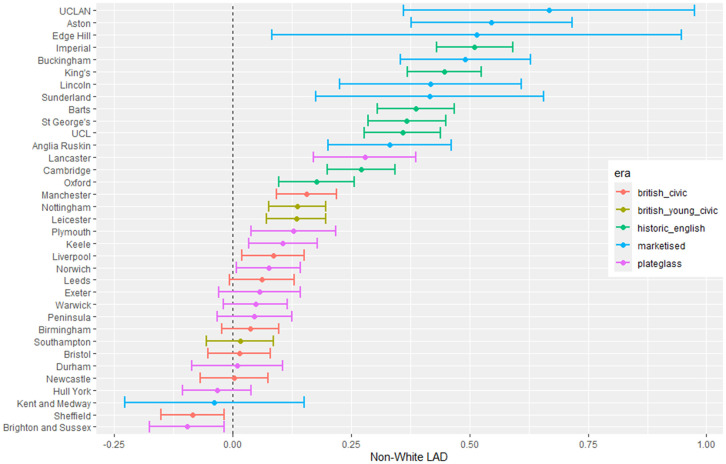
Medical school interaction effects for number of students with increasing non-white ethnicity of home local authority (non-white LAD).

**Fig 5:**
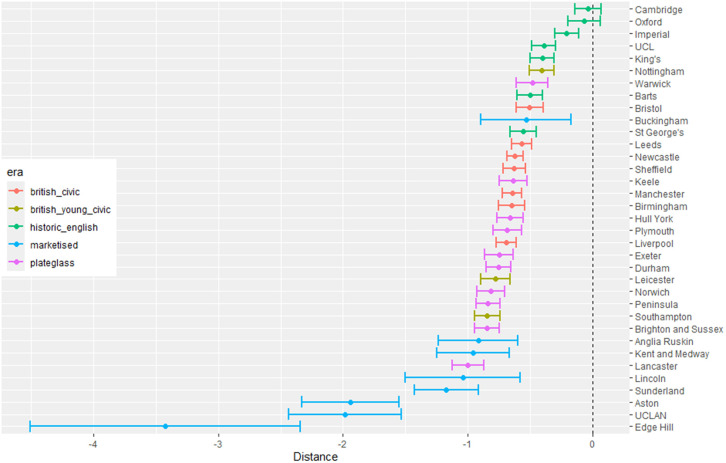
Medical school interaction effects for number of students with increasing distance from home to medical school.

**Fig 6:**
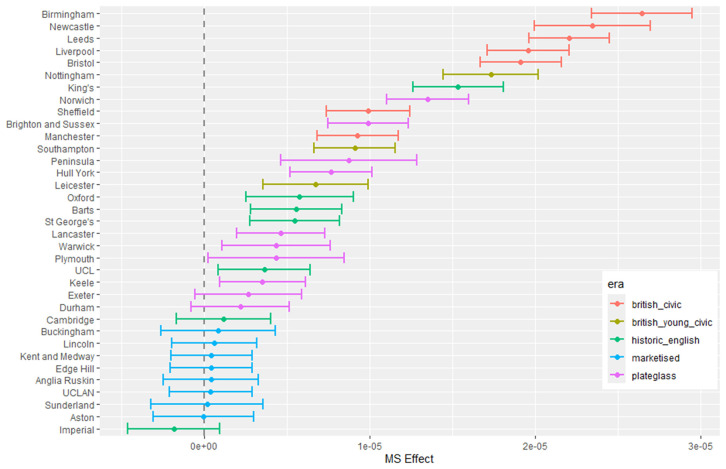
Medical school main effect (MS effect) for female minus male proportion model.

**Fig 7:**
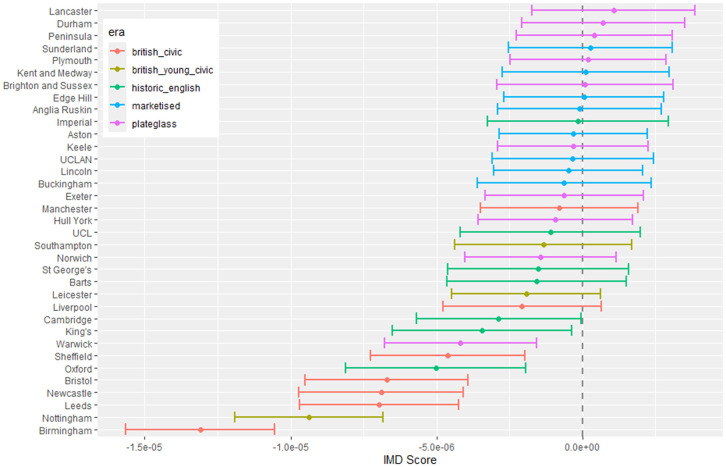
Medical school interaction effects for female minus male proportion model with increasing deprivation index (IMD score).

**Fig 8:**
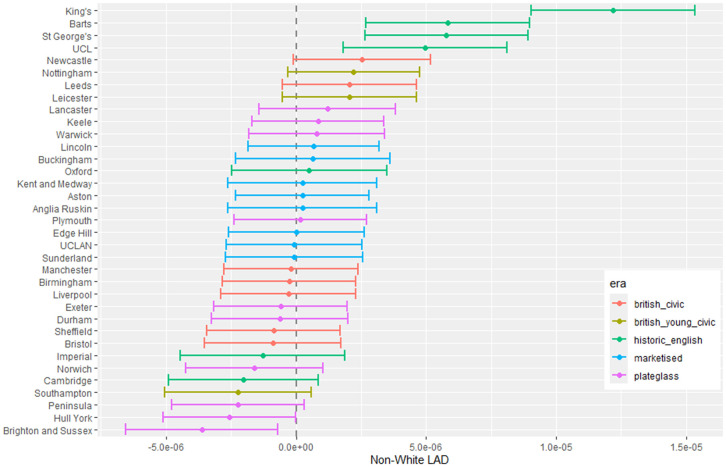
Medical school interaction effects for female minus male proportion model with increasing non-white ethnicity of home local authority (non-white LAD).

**Fig 9:**
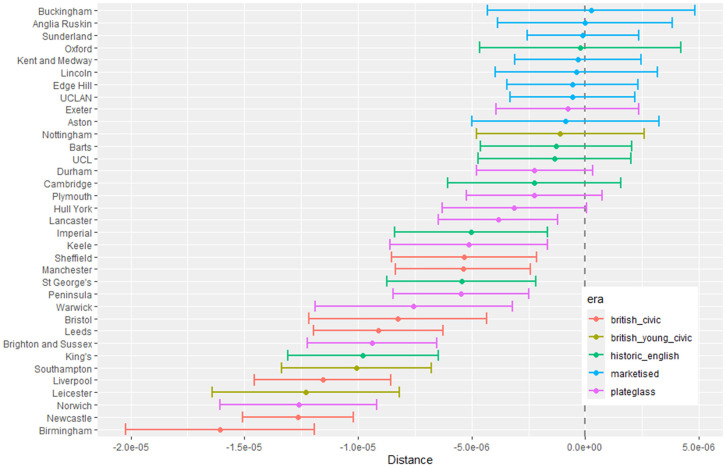
Medical School interaction effects for female minus male proportion model with increasing distance of home local authority.

**Fig 10:**
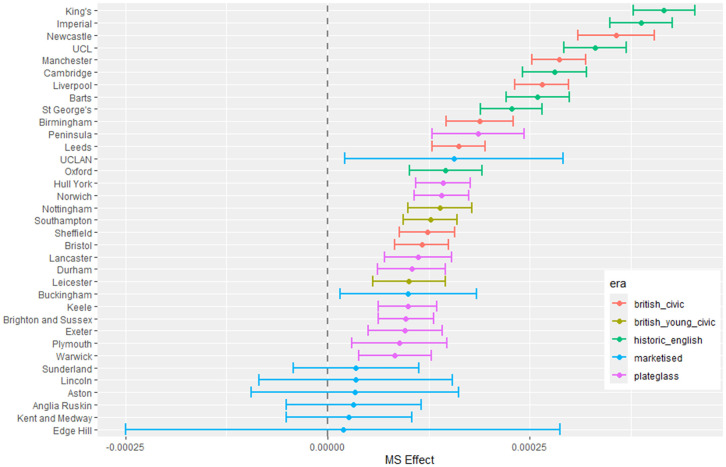
Medical school main effect (MS Effect) for non-white minus white proportion model.

**Fig 11:**
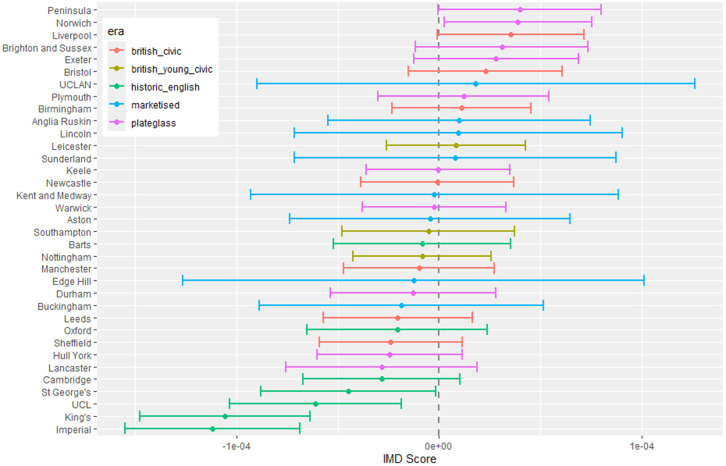
Medical school interaction effects for non-white minus white proportion with increasing deprivation index (IMD score).

**Fig 12:**
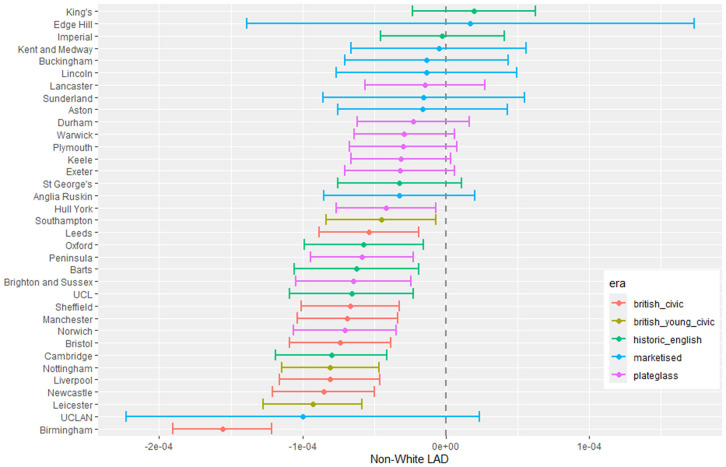
Medical school interaction effects for non-white minus white proportion with increasing non-white ethnicity of home local authority (non-white LAD).

**Fig 13:**
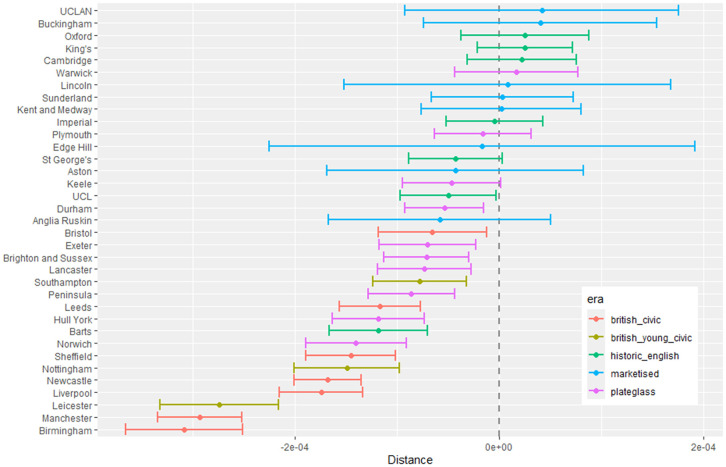
Medical school interaction effects for non-white minus white proportion with increasing distance of home local authority.

The regression parameters are either main effects of a single variable or interaction effects of a variable across medical school or foundation area. For an interaction effect, a positive value indicates that the response is increasing as the variable increases, and vice versa.

A vertical dashed line indicates zero on the parameter scale. If the confidence interval crosses this zero line, we cannot make any confident inference of its influence on the attendance to that medical school. Large bars tend to come from smaller medical schools since smaller counts of students result in relatively larger uncertainties in the Poisson model.

Bars are coloured according to the medical school era classification (ERA_*j*_ in Table 1).

#### 3.1.1 Total number of students.

Figs 3 to 5 show how medical school cohort composition varies according to local authority variables and distance from LAD to medical school. Fig 3 shows the pattern with respect to deprivation index (IMD), with all medical schools appearing to contain lower numbers from LADs with high IMD scores (the most deprived areas).

The pattern with respect to students who come from LADs with a higher proportion of non-white ethnicity in Fig 4 shows that some medical schools have more students from these LADs. This effect is largest in most of the marketised and historic English medical schools. Fig 5 shows the rate of change of number of students going to nearly all medical schools decreases with distance, except for Oxford and Cambridge. The newer marketised schools show the largest negative parameters, indicating more students come from more local populations.

#### 3.1.2 Female student proportion difference.

Figs 6 to 9 show the results of modelling female proportion minus male proportion. Fig 6 models the broad difference in gender ratio across medical schools, where the raw data values range from around 50% to 64%. The other figures in this group explain how gender differences relate to the explanatory variables with this main difference already considered.

Fig 7 shows little difference across most medical schools with respect to the deprivation index of the LAD, except where most of the British civic schools appear with low parameter values, indicating fewer female students coming from more deprived areas.

Fig 8 shows little difference in the gender proportion across all medical schools related to students from non-white LADs except for four of the London schools which show more female students coming from areas with a higher non-white population. At the other end of the chart, Brighton and Hull York perhaps show the opposite behaviour, with fewer women from higher non-white population LADs.

The greatest variation in female students across medical schools is seen in Fig 9 with the distance parameters. At the bottom of the chart, medical schools such as Birmingham, Newcastle and Norwich who have fewer female students as distance to LAD increases.

#### 3.1.3 Non-white student proportion difference.

Figs 10 to 13 show the fitted parameters for the model of non-white proportion minus white proportion. Fig 10 reflects the difference in ethnicity composition in individual medical schools which varies in the data from 20% to 60%.

Fig 11 shows little variation in ethnicity across medical schools with IMD except for most of the historic English schools appearing with larger negative parameter estimates, which show they have fewer non-white students from areas with higher deprivation in their cohorts. Fig 12 shows some difference across medical schools with respect to the non-white ethnicity of LAD, although there is not a lot of variation between schools. Birmingham stands out as having fewer non-white students from non-white LADs. Fig 13 shows most of the British civic and British young civic schools have fewer non-white students with distance from the school. Most of the London and historic schools have a distance parameter indistinguishable from zero showing no effect on non-white inclusion in student cohorts with distance.

### 3.2 Transition from medical school to foundation school

**Fig 14:**
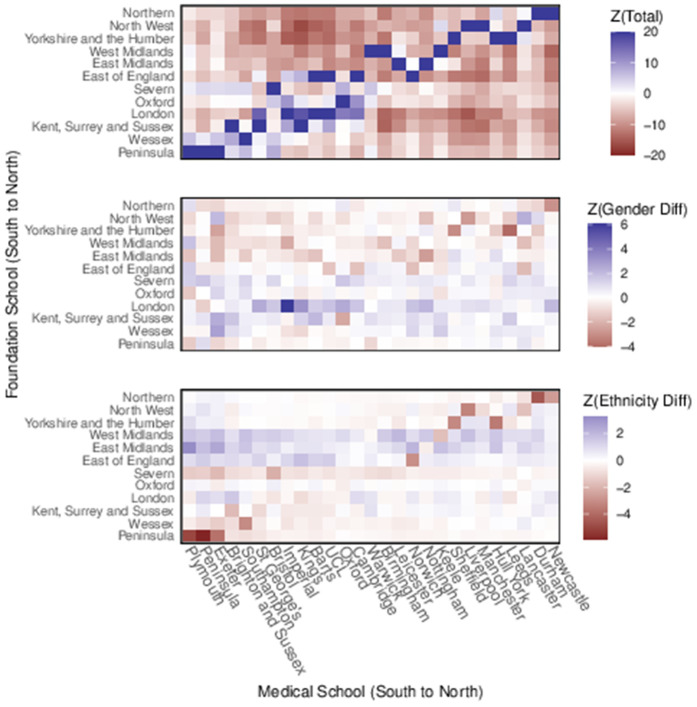
Medical school to foundation school response data ordered by south-north location; Top: Total number of students; Middle: Gender difference (F-M); Bottom: Ethnicity difference (white-non-white). Values Scaled and Centred.

Fig 14 is the equivalent of Fig 2 but for medical school to foundation training movements, with medical schools and foundation areas ordered from south to north. A very dark blue diagonal in the top panel shows strong evidence for students remaining close to their medical schools for foundation training.

There is much less structure in the gender and ethnicity panels. One observation is that the horizontal line for the London foundation area in the middle panel is excessively blue, indicating that foundation area taking on more female than male students from medical schools nationwide. In the ethnicity panel, both East and West Midlands foundation areas are bluer than others across the medical schools, showing more ethnically white students taking that route than expected. There may also be an effect of more ethnically non-white students remaining at the extreme north and south of the country given the darkest reds in the bottom left (south) and top right (north) of the graphic.

Outputs from each of the three models relating to movement from medical school to foundation school consist of a plot showing parameter estimates and ±2 standard error bounds of the discrete “distance” parameter (*s*_*ij*_) and London-related movement parameter (*L*_*ij*_), and a chart of parameter estimates for the interaction of medical school with competition ratio of the foundation area. These are coloured by medical school era classification.

#### 3.2.1 Total number of students.

**Fig 15:**
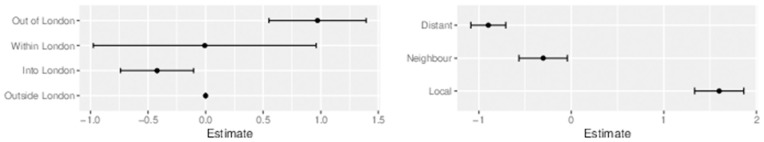
Main effects of the London-related parameter and the discrete distance parameter, for the total number of students model.

Fig 15 shows the parameter estimates and ±2 standard error measures for the parameters relating to pairs of medical school and foundation schools. The left panel shows more movement out of London compared to both non-London movement and into London. Movement into London is significantly lower than the baseline of non-London related movement, indicating that fewer students are allocated places in the London foundation school from medical schools outside London than expected. The right panel shows the significant preponderance of local moves to the coterminous foundation school over moves to the neighbouring area or other area. This indicates low mobility of medical students onward from medical school.

**Fig 16:**
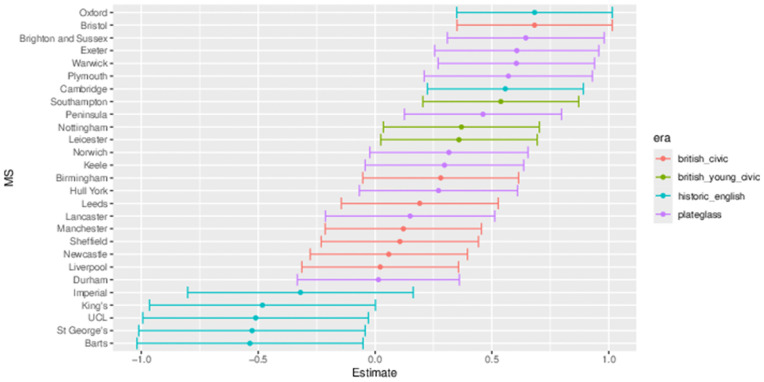
Medical school interaction effects for number of students with increasing foundation area competition ratio.

Fig 16 shows some significant difference from expected movements depending on the medical school and competition ratio of the foundation area. All the London schools group together at the bottom of the graph, with four having significantly negative parameter estimates showing fewer students going to competitive foundation areas than average, with the fifth London school, Imperial, being not far off significant as well. At the top of the graph schools including Oxford, Bristol, Brighton and Sussex, down to Nottingham have significant positive parameter estimates. More students from these medical schools are allocated training places in these more competitive foundation schools.

#### 3.2.2 Female gender proportion difference.

**Fig 17:**
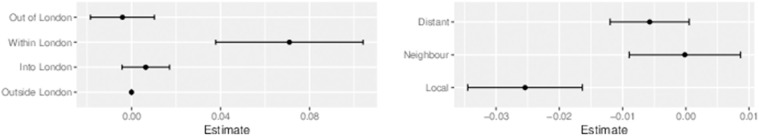
Main effects of the London-related parameter and the discrete distance parameter for the gender difference model.

Fig 17 shows the distance-related parameter estimates for the difference in proportions of women over men moving from medical school to foundation school. The left panel shows an excess of women staying within London, and the right panel shows a significantly lower proportion of women remaining in their local area after medical school training. This is in contrast to the left panel since all “within London” moves are also “Local”, implying even less local movement outside of London.

**Fig 18:**
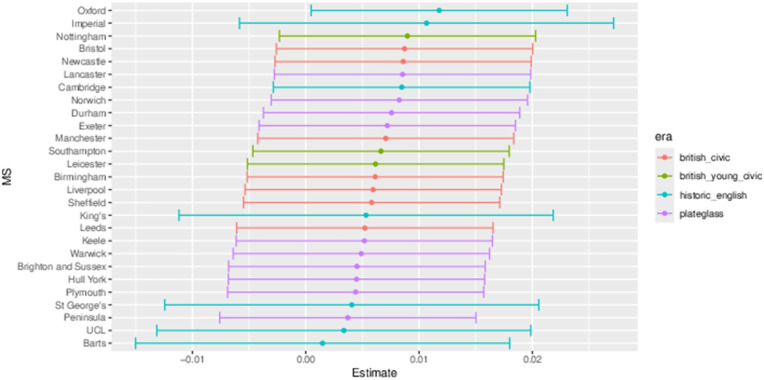
Medical school interaction effects for gender difference with increasing foundation area competition ratio.

Fig 18 shows the parameter estimates for competition ratio for each medical school. The standard error intervals for these are large and therefore it is not possible to ascertain any significant difference across them. Although nearly all the standard error envelopes contain zero, they all have a positive point estimate, indicating some evidence that relatively more female students are getting into more competitive foundation school areas.

#### 3.2.3 Non-white ethnicity proportion difference.

**Fig 19:**
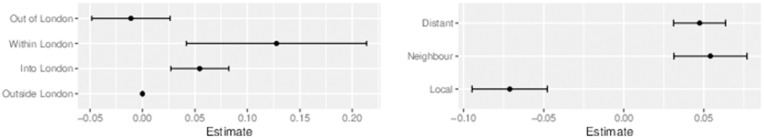
Main effects of the London-related parameter and the discrete distance parameter for the ethnicity difference model.

Both panels of Fig 19 are comparable to Fig 17, showing a higher proportion of non-white students remaining in London, while there is a contrasting low proportion difference effect of students remaining local over the country. Compared to the baseline of non-London related movement, all other moves to London are significantly elevated, especially the within-London parameter, showing a tendency to remain in London once admitted to a London medical school.

**Fig 20:**
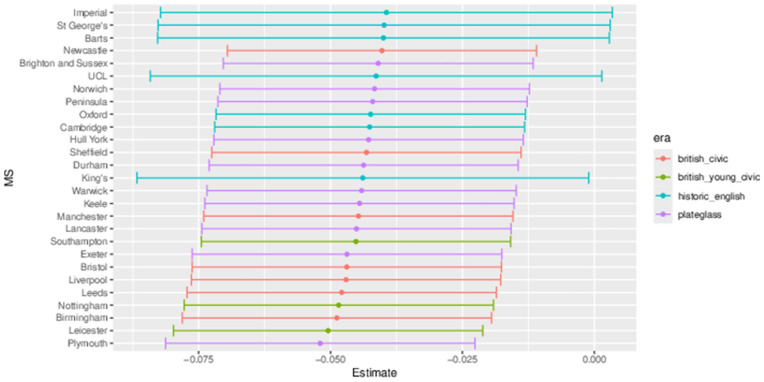
Medical school (MS) interaction effects for ethnicity difference with increasing foundation area competition ratio.

Fig 20 shows no significant difference across the medical schools with regards to their competition ratio. However in contrast to Fig 18 these values are nearly all less than zero, indicating proportionally fewer non-white students going to the more competitive foundation schools.

## 4 Discussion

The findings from these statistical analyses show that medical schools predominantly select disproportionately fewer students from more deprived areas, and this is particularly pronounced at some medical schools, which we categorised as historic English schools. Medical school student cohorts are more likely to have students from nearby LADs, though the strength of this effect varies considerably between medical schools. Marketised schools are more likely to have students originally based locally, but as the amount of data for these schools is smaller, confidence interval estimates are wider. From these analyses we have highlighted how, while medical students often choose to go to a medical school in their local area (the “distance effect”) the medical school cohorts still contain disproportionately fewer students from more socio-economically deprived areas, even when accounting for distance. The distance effect is less pronounced for some schools, showing that students are travelling further to attend, e.g., Oxford and Cambridge.

Some findings can partly be explained by other factors; Birmingham, for example, has the largest and most noticeably different in its values with regards to distance, and ethnicity. This is partly explained by: its geography (relatively central to other locations in England); the large size of the LAD in comparison with other LADs; its relatively large size as a medical school; the overall ethnic diversity of the communities that live in Birmingham. This explanation shows that the application of statistical modelling for future workforce planning always needs to be interpreted with appropriate accounting for complexity. All medical schools have more female students than male students, as per national trends, and there is a higher proportion of students from non-white backgrounds than the national population average.

Medical schools admissions policies are managed by each medical school, and we do not seek to explore or critique these policies here. Instead we seek to explicate some of the relevant differences between medical schools considering implications for both social mobility, and diversity in the future medical workforce.

When following students from medical school into foundation training, the distance effect continues to have an impact. Findings indicate low mobility overall, with students staying in the same region as their medical schools. There are indications that proportionally, women and non-white students move further. The different behaviour of these groups needs to be explained by factors not visible in these analyses.

Analysis of the impact of competition ratios on the allocation of foundation places for students from different medical schools can be best explained by understanding of the context. London, as defined in our analysis, is the most competitive foundation school. More applicants want to work there than there are training places, and there are more medical students than foundation training places in London. There is, therefore, a higher likelihood that students will have to leave London and go elsewhere in the UK to continue their career. Under the previous foundation programme allocation system, candidates were ranked by decile according to their educational performance measure and a situational judgement test. The top decile from each medical school were more likely to be offered their first choice foundation school. This system changed in 2024 to a preference-based allocation system, but the data presented here were collected prior to this change and so the impact on the London schools (with negative parameter estimates) is visible [36].

Strengths of this study include the application of statistical modelling to a large dataset focused on medical students and doctors, using the IMD as a proxy for socioeconomic status to understand the relationship between deprivation and medical school admissions and graduates. However, there are acknowledged problems with the IMD as a measure, as it relies on postcode data not individual circumstances. As this is not individual level modelling, we were not able to add in other proxy measures such as parental occupation or other recognised additional measures to supplement our understanding of deprivation. As education is a domain within the IMD (the Education, Skills and Training Deprivation Domain contains outcomes for children and young people including key stage 2 and key stage 4 attainment, secondary school absence, staying on in education and entry to higher education, as well as adults skills including adults with no or low qualifications and English language proficiency), we did not model other educational outcomes available in UKMED data separately. Limitations also include the focus on England; due to the differences in IMD classification measurement techniques across the devolved nations, we could only apply this model to England, and English medical schools. Given the differences in higher education and health policy across the four nations, alongside the permeability of these boundaries in practice for students going to university, it would have been interesting to look across the four nations to see if these differences impacted on observed patterns.

GMC reporting on the workforce as a whole suggests that there are more female doctors than male doctors, a greater proportion of people from diverse ethnic backgrounds than in the general population, and a relatively high reliance on recruiting doctors from outside the UK to meet population needs [37,38]. Our analysis of UKMED data suggests similar trends will continue in terms of gender and ethnicity.

Previous analyses of the UKMED data have focused on selection and admissions data, attainment, demographic factors (gender, ethnicity, widening participation status) [39–42]. There has also been interest in the relationship between educational measures and outcomes, explored elsewhere [43]. Interest in movement patterns of doctors’ careers has led to several analyses of subsets of available data [40,44]. These analyses have been influential in shaping understandings informing policy. Data have previously suggested that medical students tend to go to medical school close to their home location, and then train close to home location, generally seen as within 50 miles of their initial starting location [40,44]. Most of these variables have been found significant in the movements of future doctors in past literature [45–48]. Previous analyses of the transition to foundation training have concluded that there is a statistically significant difference between the knowledge and skills of doctors (as measured by the three metrics defined by the authors in this study) entering the Foundation Programme in different foundation schools [49]. Other studies have used publicly-available data to identify and understand differences between medical schools [50]. In reflecting on the complexity of unpicking the diversity of influences on medical careers, the paper by McManus et al identified that institutional histories related to outcomes for students [50]. Our paper expands beyond thinking about individual schools to consider a broader categorisation of insitutional types in relation to the detailed data in the UKMED dataset.

Our findings contribute to longstanding interest in understanding the observed social gradient in medicine [51]. As attention has focused on widening participation in medicine, it might be expected that improvements over time could be observed. The recent Medical Schools Council report, Fostering Potential, suggests progress has been made in overcoming inequalities [52]. In contrast, analysis from the educational charity the Sutton Trust aimed to compare the five newest medical schools with previously established ones, with a view to examining social mobility. This report concluded that school type, parental occupation, IMD and region all impact on whether or not a student is offered a place at medical school [23]. Our results align with these findings, showing that despite moves towards equity and widening participation in medicine, this has not yet been achieved.

There are two main implications of this research. First, that while greater diversity of the future medical workforce in terms of gender and ethnicity has been achieved, there is less evidence that widening participation initiatives have had significant effect on changing the landscape around inclusion of students from more deprived areas. Second, that there is still variation between medical schools in terms of the composition of student cohorts, and this relates to socio-economic deprivation. While, as above, there is individual-level university control for admissions policies, unless sector-wide changes are agreed, there is a risk that students from a socio-economically deprived background will remain disproportionately excluded. A second risk is that there is, inadvertently, the creation of a ‘two-tier’ system, in which some medical schools work to educate more diverse students, and others remain a closed door to students from socio-economically deprived areas. Perceptions that this is occurring, with medical schools influencing outcomes for students, can be observed in qualitative data collected as part of the wider study on which this paper is based, which are reported elsewhere [53].

While admissions processes for medical schools are not directly linked to healthcare needs in a particular area, the observed ‘distance effect’ shows that they have a potential impact on recruitment of doctors in the long term. Our findings also have implications for current workforce planning policies. From our analysis, it is clear that the stated ambitions to increase the number of medical student places in England will not be sufficient to address inequalities. Expansion of student numbers will need to be accompanied by targeted measures to improve access to medicine for students from socio-economically deprived backgrounds. Without such efforts, there is a risk that expansion will reinforce existing patterns of exclusion rather than addressing them. Future research should focus on considering the impacts of the longitudinal relationships between medical school attended and medical career outcomes.

## Supporting information

S1 File Information. Medical school classification scheme. (PDF)

S2 Table Model summary outputs. (PDF)
