# Correction: Where do they come from and where do they go: Understanding the relationship between deprivation and the geographical journeys of trainee doctors in England

**DOI:** 10.1371/journal.pone.0352603

**Published:** 2026-06-25

**Authors:** Barry Rowlingson, Peter Diggle, Liz Brewster, Michael Lambert

There are errors in the Funding statement. The correct Funding statement is as follows: This study is funded by the National Institute for Health and Care Research (NIHR) (NIHR HSDR 134540). The views expressed are those of the author(s) and not necessarily those of the NIHR or the Department of Health and Social Care. The funders had no role in study design, data collection and analysis, decision to publish, or preparation of the manuscript.
