# Supplementary material for: Where do they come from and where do they go: Understanding the relationship between deprivation and the geographical journeys of trainee doctors in England
Source: PLoS One. 2026 Mar 30;21(3):e0345301. doi: 10.1371/journal.pone.0345301 (PMC13035342; doi:10.1371/journal.pone.0345301)
Supplement: S1 File — (PDF) [file pone.0345301.s001.pdf]

Supporting Information

Medical School Classification Scheme

'Civic', 'plateglass' and 'marketised' refer to types of university and their development over time. These are recognised labels used in the literature on the history of education which reflect their origins and style. We have applied these terms based on the university that the school is a part of.

English and Scottish Historic:

Established before 1900 in hospitals designed for medical research and teaching as well as service provision, creating the model of single-site medical/university teaching. In London, there have been many mergers over time.
Cambridge, Charing Cross, Guy's Hospital, King's College, London Hospital, London (Royal Free) School, Middlesex, Oxford, St Bartholomew's, St George's, St Mary's Hospital, St Thomas', University College Hospital Westminster. Aberdeen, Edinburgh, Glasgow, St Andrew's.

British Civic:

The label 'civic' has a much broader contemporary meaning in relation to higher education, but historically it refers to universities established in large provincial centres from the 1860s to the 1900s which progressively obtained degree granting powers separate from the University of London. They are often called 'redbrick', and were 'civic' in their focus on providing higher education in their communities, often with a strong applied focus. Most provincial medical schools were established prior to this, but became attached to universities as they obtained charters and degree granting powers from the 1890s to 1910s.

Located in new universities around the turn of the twentieth century, in large cities, often incorporating existing hospital medical schools so providing single-site teaching.
Belfast, Birmingham, Bristol, Cardiff, Leeds, Liverpool, Manchester, Newcastle, Sheffield

British Young Civic:

Established between 1948-63 as university colleges in large cities became universities in their own right. Authorised to create new medical schools by the 1968 Royal Commission on Medical Education and again designed for single-site teaching.
Dundee, Leicester, Nottingham, Southampton

Plateglass:

'Plateglass' refers to the common architectural style of newer universities established in the 1960s as part of the 1963 Robbins Report. This was in contrast to 'redbrick' 'civic ones, and were established in large towns rather

than cities without a university catering for greater access to higher education, often with newer degree courses. Medical schools were not founded until the 1997 Medical Workforce Standing Advisory Committee's third report's recommendations. Mostly in sites where single large hospitals had the existing scope to be used for teaching but the hospitals with which they were associated were not designed purposefully for teaching and research.

Brighton and Sussex, Durham, Hull York, Keele, Lancaster, Norwich, Peninsula (Exeter), Peninsula (Plymouth), Swansea, Warwick

Marketised

Institutions which became universities following the 1992 Further and Higher Education Act, mainly in large English towns. 'Marketised' is a common labels for universities created from a range of polytechnical and other colleges after universities were marketised in 1992 under the Further and Higher Education Act. The label 'post-92' attracts considerable stigma and does not encompass universities established in a later wave from 2002-08. Using 'marketised' reflects their historical origins without drawing upon 'post-92' and its negative connotations. Medical schools were associated with the 2017 Department of Health expansion of undergraduate student places.

Anglia Ruskin, Aston, Bangor, Brunel, Buckingham, Chester, Cumbria University (Pears), Edge Hill, Kent Medway, Lincoln, Magee (Ulster), Sunderland, Three Counties (Worcester), University of Central Lancashire
