# Supplementary material for: Where do they come from and where do they go: Understanding the relationship between deprivation and the geographical journeys of trainee doctors in England
Source: PLoS One. 2026 Mar 30;21(3):e0345301. doi: 10.1371/journal.pone.0345301 (PMC13035342; doi:10.1371/journal.pone.0345301)
Supplement: S2 Table — (PDF) [file pone.0345301.s002.pdf]

Supporting Information

Model Summary Outputs

Tables 1 to 6 show, for each of the six models, the changes in degrees of freedom, deviance and related statistics when adding successive terms to a null model. Small chi-squared statistic values are truncated at 1e-06.

Note that all parameters in the home to medical school models improve the model by reducing the deviance a large amount judged by the likelihood ratio test of model comparison.

Some parameters in the medical school to foundation area models do not reduce the deviance significantly and could reasonably be removed from the model for parsimony but we leave them in to demonstrate this in the analysis in the text.

	df	deviance	resid.df	resid.dev	Pr(Chisq)
(Constant)			11095	662063	
MedSchool	35	639640	11060	22422	<1e-06
Distance	35	9837	11025	12586	<1e-06
LAD Ethnicity	35	626	10990	11969	<1e-06
IMD Score	35	1318	10955	10641	<1e-06

Table 1: Home to Medical School, Total Number Model

	df	deviance	resid.df	resid.dev	Pr(Chisq)
(Constant)			8857	7.1e-06	
MedSchool	35	1.3e-06	8522	5.7e-06	<1e-06
Distance	35	4.0e-07	8487	5.3e-06	<1e-06
LAD Ethnicity	35	1.0e-07	8452	5.3e-06	<1e-06
IMD Score	35	1.0e-07	8417	5.1e-06	<1e-06

Table 2: Home to Medical School, Gender Difference Model

	df	deviance	resid.df	resid.dev	Pr(Chisq)
(Constant)			8857	0.00122	
MedSchool	35	3.88e-04	8522	0.000835	<1e-06
Distance	35	8.35e-05	8487	0.000752	<1e-06
LAD Ethnicity	35	3.61e-05	8452	0.000716	<1e-06
IMD Score	35	8.00e-06	8417	0.000708	<1e-06

Table 3: Home to Medical School, Ethnicity Difference Model

	df	Deviance	Resid. Df	Resid. Dev	Pr(Chisq)
(Constant)			324	1330.36	
Discrete Distance	3	883.23	321	447.13	<1e-06
London Move	3	31.32	318	415.81	<1e-06
Comp. Ratio	27	78.25	291	337.56	<1e-06

Table 4: Medical School to Foundation Area, Total Number Model

	df	Deviance	Resid. Df	Resid. Dev	Pr(Chisq)
(Constant)			324	0.1138	
Discrete Distance	3	0.00165	321	0.112	0.112
London Move	3	0.0289	318	0.083	<1e-06
Comp. Ratio	27	0.00289	291	0.080	0.998

Table 5: Medical School to Foundation Area, Gender Difference Model

	df	Deviance	Resid. Df	Resid. Dev	Pr(Chisq)
(Constant)			324	0.888	
Discrete Distance	3	0.255	321	0.633	<1e-06
London Move	3	0.0320	318	0.601	0.0006
Comp. Ratio	27	0.0603	291	0.541	0.215

Table 6: Medical School to Foundation Area, Ethnicity Difference Model
